# Hypervirulent *Klebsiella pneumoniae* induces liver abscess by promoting neutrophil extracellular trap formation through NLRP3 inflammasome activation

**DOI:** 10.1128/spectrum.02376-25

**Published:** 2026-05-18

**Authors:** Qian Ou, Meixia Cheng, Liming Fan, Jingyi Zou, Kaiwen Wang, Ziwen Xie, Weiyu Jiang, Keyi Gong, Jiadong Wang, Ziyan Jiang, Xinyu Zhu, Qiang Wang, Tiejun Zhao, Jiaqi Fang

**Affiliations:** 1 Department of Clinical Medicine, Hangzhou City University School of Medicine 728194, Hangzhou, People's Republic of China; 2 Department of Basic Medical Sciences, Zhejiang University School of Medicine 26441, Hangzhou, People's Republic of China; 3 Zhejiang University School of Medicine 26441, Hangzhou, People's Republic of China; 4 The Second Clinical Medical College, Zhejiang Chinese Medical University 70571, Hangzhou, People's Republic of China; 5 Department of Clinical Laboratory, The Second Affiliated Hospital of Zhejiang Chinese Medical University 587400 , Hangzhou, Zhejiang, People's Republic of China; Geisel School of Medicine at Dartmouth, Lebanon, New Hampshire, USA

**Keywords:** hypervirulent *Klebsiella pneumoniae*, liver abscess, NETosis, NLRP3 inflammasome

## Abstract

Hypervirulent *Klebsiella pneumoniae* (hvKP) has spread globally, causing severe liver abscesses. Neutrophil extracellular trap formation (NETosis) is an essential defense mechanism of neutrophils. However, the role of pathogenicity and molecular mechanism of liver abscesses induced by hvKP on neutrophil extracellular trap formation remain unclear. Here, we revealed abscesses and severe neutrophil infiltrates in the livers of hvKP-infected mice, which can be compared to the mice infected with classical *Klebsiella pneumoniae* (cKP). hvKP infection significantly increased reactive oxygen species production, NETosis, and apoptosis-associated speck-like protein containing a CARD foci formation in neutrophils compared to cKP. In addition, hvKP infection upregulated the mRNA levels and protein expression levels of key NETosis-associated molecules (Caspase-1, MPO, PAD4, and CitH3) relative to cKP, whereas NLRP3 expression remained unchanged in neutrophils. Furthermore, hvKP infection also maintained a higher survival rate within neutrophils compared with cKP and promoted neutrophil extracellular trap formation in an NLRP3 inflammasome activation and PAD4-dependent manner *in vitro* and *in vivo*. Moreover, hvKP infection promoted the production of IL-1β, IL-18, and TNF-α from patients’ serum compared to cKP. Our findings demonstrate that hvKP induces NETosis formation, which might contribute to tissue injury rather than bacterial clearance in mice. This suggests a pathogenic role of NETosis in hvKP-mediated infection and identifies a potential target for therapeutic intervention.

**IMPORTANCE:**

Hypervirulent *Klebsiella pneumoniae* (hvKP) has emerged as a globally prevalent pathogen that triggers severe liver abscesses, posing a substantial threat to public health. Neutrophil extracellular trap formation (NETosis) serves as a core innate immune defense against invading microbes. However, how hvKP manipulates NETosis to drive liver abscess progression remains undefined. This study demonstrates that hvKP induces more severe hepatic lesions and neutrophil infiltration than classical *Klebsiella pneumoniae*, while enhancing reactive oxygen species production, NETosis, and the expression of key NET-related proteins in neutrophils. Additionally, hvKP infection elevates pro-inflammatory cytokine levels in patient serum. These findings provide a novel insight into hvKP-mediated innate immune evasion and the pathogenesis of liver abscesses induced by hvKP.

## INTRODUCTION

*Klebsiella pneumoniae* is a common opportunistic pathogen that frequently causes nosocomial infections or community-associated infections, including pneumonia, meningitis, bloodstream and urinary tract infections, liver abscesses, and endophthalmitis (1, 2). Infections are more common in the Asian Pacific Rim but are occurring globally (3, 4). Hypervirulent *Klebsiella pneumoniae* (hvKP)-induced liver abscesses have emerged during the past two decades, which is an evolving pathotype that is more virulent than classical *Klebsiella pneumoniae* (cKP) (5, 6).

The characteristic pathological changes of acute liver abscess are neutrophil infiltration (7, 8). It is well-known that neutrophils are considered the immune system’s first line of defense against pathogens and first responders to endogenous irritants (9, 10). They achieve their host defense role by phagocytosing pathogens, secreting their granules full of cytotoxic enzymes, or expelling neutrophil extracellular traps (NETs) during the process of NETosis (11, 12). NETs are a three-dimensional meshwork consisting of chromatin, antimicrobial components such as neutrophil elastase (NE) and myeloperoxidase (MPO), and the nicotinamide adenine dinucleotide phosphate (NADPH) oxidase involved in producing reactive oxygen species (ROS) (13, 14). It has been reported that hvKP displays a significantly higher level of resistance to neutrophil-mediated phagocytosis and killing in comparison to cKP strains, which are more readily retained in NETs (15).

NETosis can be induced by a wide range of substances, including bacteria, lipopolysaccharide (LPS), phorbol-12-myristate-13-acetate, viruses, etc. (13, 16, 17). The specific requirements for NET formation depend on the stimulus, but histone citrullination mediated by peptidylarginine deiminase 4 (PAD4) has been demonstrated to be an essential step in NETosis (18, 19). In neutrophils, the NLR family pyrin domain-containing 3 (NLRP3) inflammasome is the most documented as a broad sensor of cellular damage, which is activated after bacterial infection or LPS pretreatment with subsequent ATP stimulation (20–22).

Although NETs constitute one of the major antimicrobial defense mechanisms in neutrophils against invading pathogens, especially against hvKP, their influence on NETosis and NLRP3 inflammasome assembly in neutrophils or their potential role in NETosis remains elusive. In the present study, we show that hypervirulent *Klebsiella pneumoniae* promotes neutrophil extracellular trap formation by activating NLRP3 inflammasome. Our findings not only provide a novel mechanism underlying hvKP innate immune evasion but also offer new insights into the pathogenicity of pathological microbes.

## RESULTS

### Abscesses in the livers could be observed in the hvKP-induced mouse model

**Fig 1:**
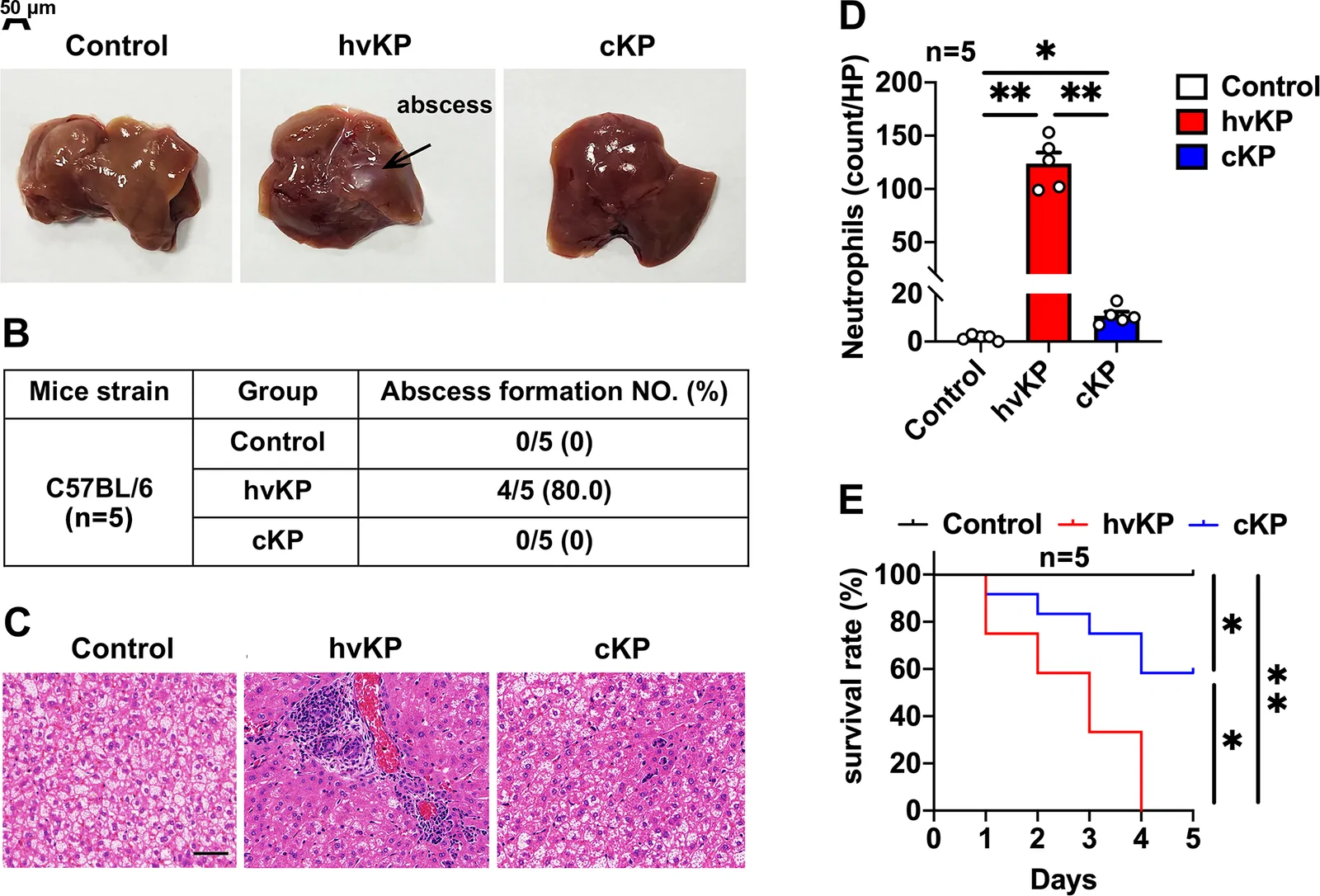
Pathogenicity of hvKP- or cKP-induced murine model. (**A**) Gross pathological changes in livers from hvKP- or cKP-induced murine models. (**B**) Percentages of abscess formation in different groups (*n* = 5). (**C**) Histological examinations of livers from mouse models. Scale bar = 50 µm. (**D**) Statistical summary of neutrophil counts per high power field (HP) in panel **C**. (**E**) Survival rate of mice infected with hvKP or cKP (*n* = 5). Data are representative of five biological repeats in panels A–E (*n* = 5). *P < 0.05 and **P < 0.01.

We infected mice with hypervirulent *Klebsiella pneumoniae* strain NTUH-K2044 and classic *Klebsiella pneumoniae* strain HS11286. Abscesses in the livers could be observed in hvKP-infected mice, and no abscess was present in livers of cKP-infected mice (Fig. 1A and B). Correspondingly, a large number of neutrophils infiltrating the livers was observed in the hvKP-infected mice compared with the cKP-infected group (Fig. 1C and D). Moreover, survival rates of the mice injected with hvKP (0%) at 5 days were significantly lower than that of cKP (60.0%) (Fig. 1E). Collectively, these results showed that the pathogenicity of hvKP was stronger than that of cKP.

### hvKP increases ROS levels in neutrophils and enhances its intracellular survival

**Fig 2:**
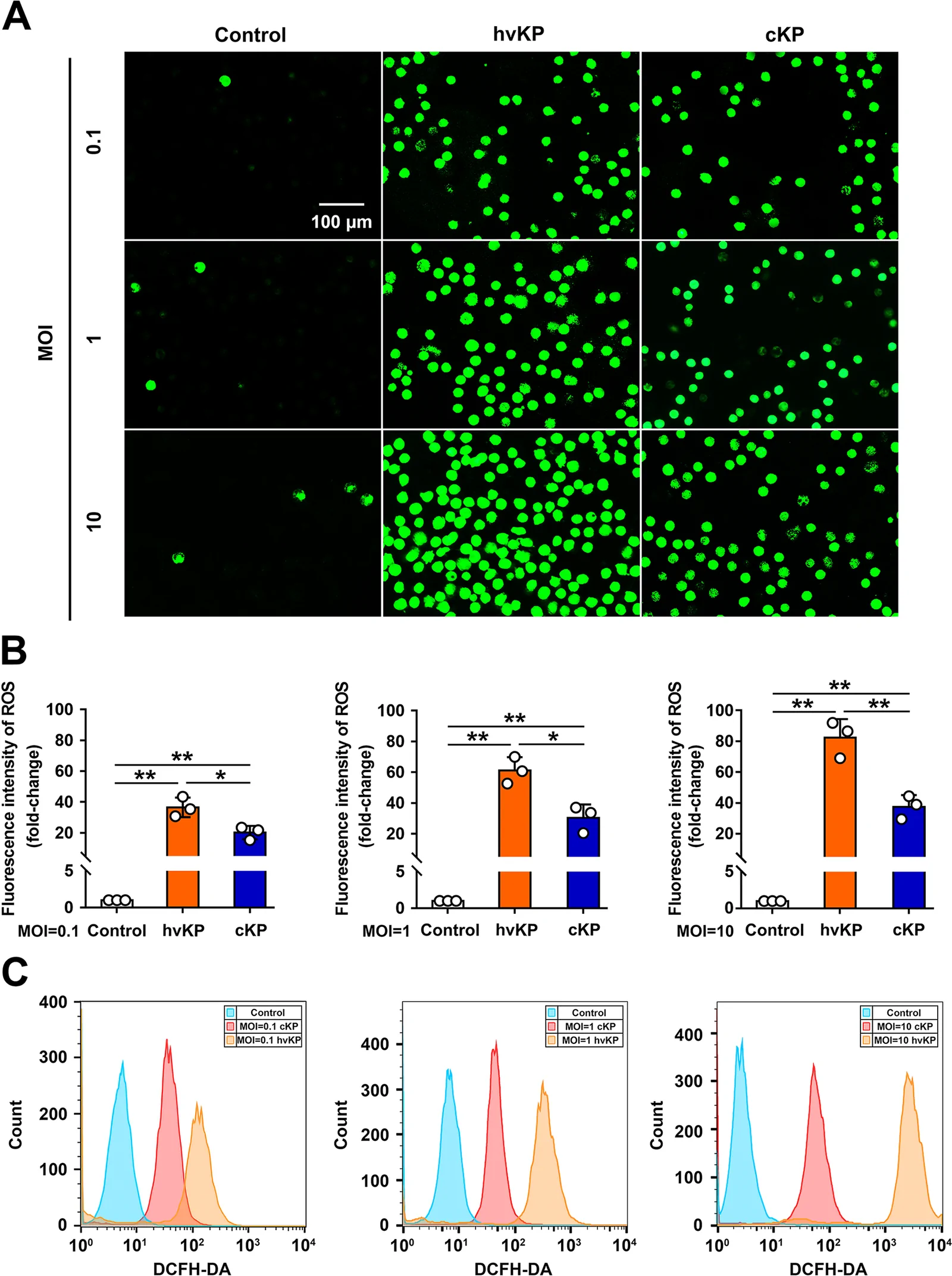
hvKP significantly accelerates the ROS levels in neutrophils. (**A**) Confocal analyses of the influence of hvKP or cKP on ROS production. Scale bar = 100 µm. Images shown are representative of three independent experiments. *n* = 3. (**B**) Fluorescence intensity (FI) of ROS was analyzed by ImageJ software. The FI reflecting ROS level in the control group was set as 1.0. *P < 0.05 and **P < 0.01. (**C**) Flow cytometry detection of the effects of hvKP or cKP on ROS production in neutrophils. Data are representative of three biological repeats in panels **A–C** (*n* = 3).

hvKP is generally associated with the hypermucoviscosity phenotype, which is typically due to the increased production of capsular polysaccharide and the presence of specific virulence genes (23). The PCR results revealed that hvKP, but not cKP, possess the virulence genes, including *magA*, *rcsA*, *rcsB*, *rmpA2*, and *rmpA* (Fig. S1A). String test results confirmed that hvKP produced a viscous string with a visual length of 58 mm, whereas cKP produced a string of only approximately 4 mm (Fig. S1B). To investigate the survival ability of hvKP or cKP in neutrophils, human peripheral blood neutrophils with a purity of 98.9% (data not shown) were infected with hvKP and cKP at different MOIs (MOI = 0.1, 1, and 10) for 4 h, respectively. The number of intracellular viable bacteria in the hvKP group was significantly higher than that in the cKP group, suggesting that hvKP is more capable of evading neutrophil killing than cKP (Fig. S2). In ROS-dependent NETosis, neutrophils produce ROS to trigger degranulation of MPO, which is then transported to depolymerized DNA when cells are infected by pathogens (24). The influence of hvKP or cKP on ROS was examined, and confocal results showed that hvKP obviously increases the production of ROS in neutrophils compared to the group infected with cKP (Fig. 2A and B). When the ROS levels were measured by flow cytometry, results that mirrored the same trend were also obtained (Fig. 2C). These data indicate that hvKP markedly promote ROS production and enhance its survival in neutrophils, probably due to the presence of virulence genes.

### hvKP promotes NETosis in neutrophils

**Fig 3:**
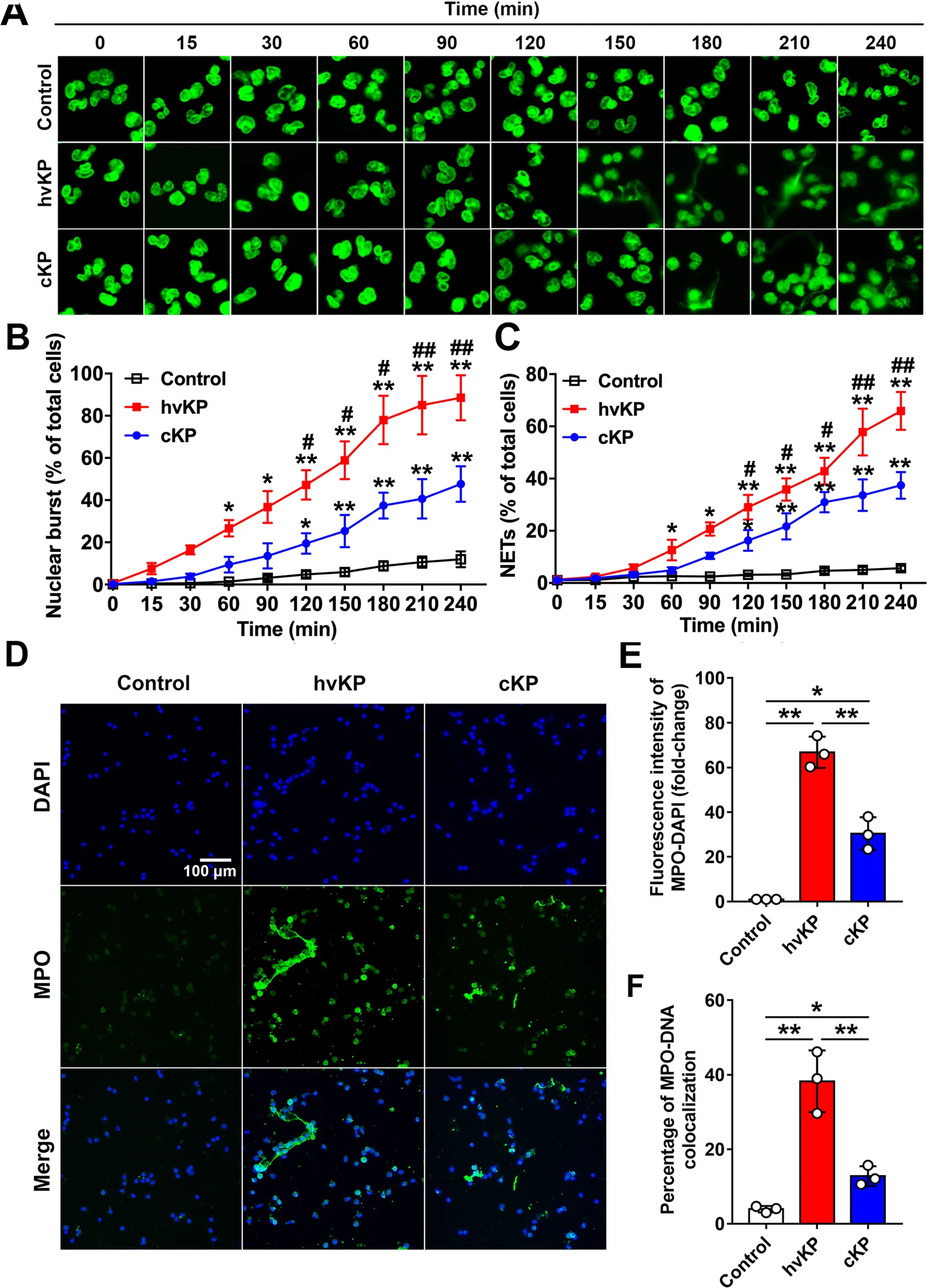
hvKP significantly promotes chromatin decondensation in NETosis. (**A**) Dynamic observation of neutrophil DNA decondensation by Sytox Green staining. Scale bar = 20 µm. (**B**) Analyses of DNA release area from experiments as described in panel A. *, #*P* < 0.05 and **, ##*P* < 0.01. *, hvKP or cKP vs control; #, hvKP vs cKP. (**C**) Percentage analyses of NETosis in total neutrophils. *, #*P* < 0.05 and **, ##*P* < 0.01. *, hvKP or cKP vs control; #, hvKP vs cKP. (**D**) Confocal observation of the influence of hvKP on ROS-dependent NETosis. Scale bar = 100 µm. (**E**) FI of co-localization in experiments as described in panel D. The FI reflecting NETosis level in the control group was set as 1.0. *P < 0.05 and **P < 0.01. (**F**) Percentage of co-localization reflecting the NETosis levels in experiments as described in panel D. *P < 0.05 and **P < 0.01. Data are representative of three biological repeats in panels A–F (*n* = 3).

It has been reported that NETosis can be induced by a wide range of substances, including bacteria, lipopolysaccharide, phorbol-12-myristate-13-acetate, viruses, etc. (13, 25). We detected NETosis by observing neutrophil chromatin depolymerization dynamically using confocal microscopy. The results demonstrated that chromatin depolymerization in neutrophils treated by hvKP was obviously promoted when compared with those in cKP-treated groups (Fig. 3A through C). MPO is one of the major antimicrobial components in the NETs, which are three-dimensional meshworks consisting of chromatin (26). Therefore, the co-localization of MPO and DNA was observed by fluorescence microscope to further confirm that hvKP stimulated NET formation more significantly than cKP (Fig. 3D through F). Consistent with its enhanced pathogenic effects on the mouse liver, hvKP also maintains a higher survival rate within neutrophils (or exposure to pre-formed NETs with or without DNase) compared with cKP (Fig. 1; Fig. S2 and Fig. S3D), demonstrating that NETosis formation might contribute to tissue injury rather than bacterial clearance in mice.

### hvKP affects gene transcription during NLRP3 inflammasome activation and NETosis in neutrophils

**Fig 4:**
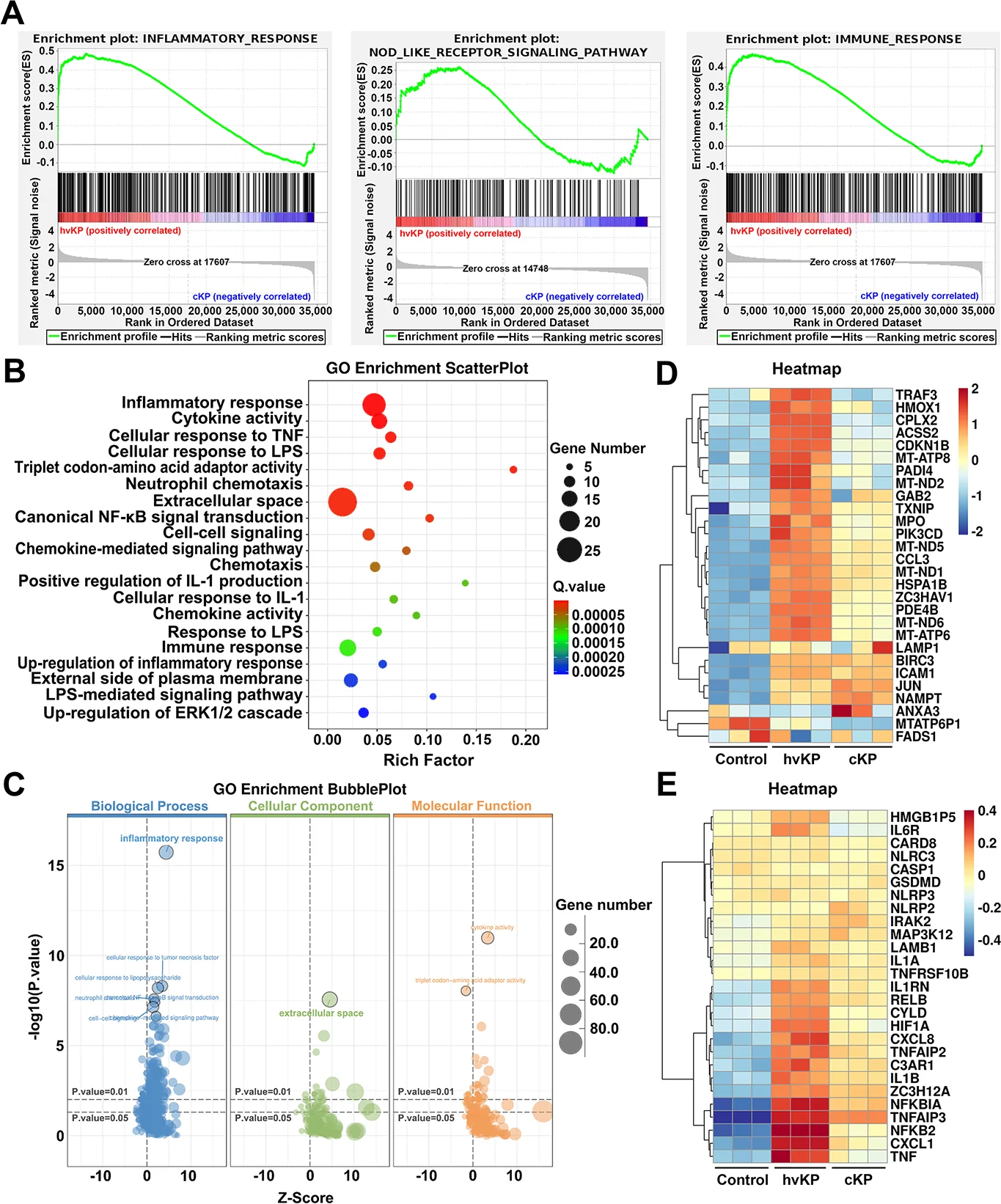
hvKP or cKP affects the transcription of genes in neutrophils. (**A**) hvKP enhances the inflammatory response, nod-like receptor signaling pathway, and immune response in neutrophils. (**B**) hvKP dramatically affects the chemotaxis and immunity- and inflammation-related genes in neutrophils. (**C**) hvKP affects the enrichment of physiological activity-related genes in neutrophils. (**D and E**) NETosis formation and NLRP3 inflammasome activation transcripts were significantly upregulated in hvKP-infected neutrophils compared with those from cKP-infected. Data are representative of three biological repeats in panels A–E (*n* = 3).

To determine the transcriptional activity of NETosis-related genes, we conducted a genome-wide transcriptomics analysis of neutrophils treated with hvKP or cKP. All the transcriptomics data used in this study were uploaded to ScienceDB (https://www.scidb.cn/en), and the accession CSTR number is 31253.11.sciencedb.34045. hvKP enhanced the inflammatory response, nod-like receptor signaling pathway, and immune response in neutrophils compared to cKP (Fig. 4A). In addition, hvKP dramatically affected the chemotaxis and immunity- and inflammation-related genes in neutrophils (Fig. 4B and C). Furthermore, NETosis formation and NLRP3 inflammasome activation transcripts were significantly upregulated in hvKP-infected neutrophils (Fig. 4D through E). This implies that hvKP promoted NETosis formation by activating an inflammatory response in neutrophils.

### hvKP significantly promotes the activation of NLRP3 inflammasome and expression of NETosis-associated proteins in neutrophils

**Fig 5:**
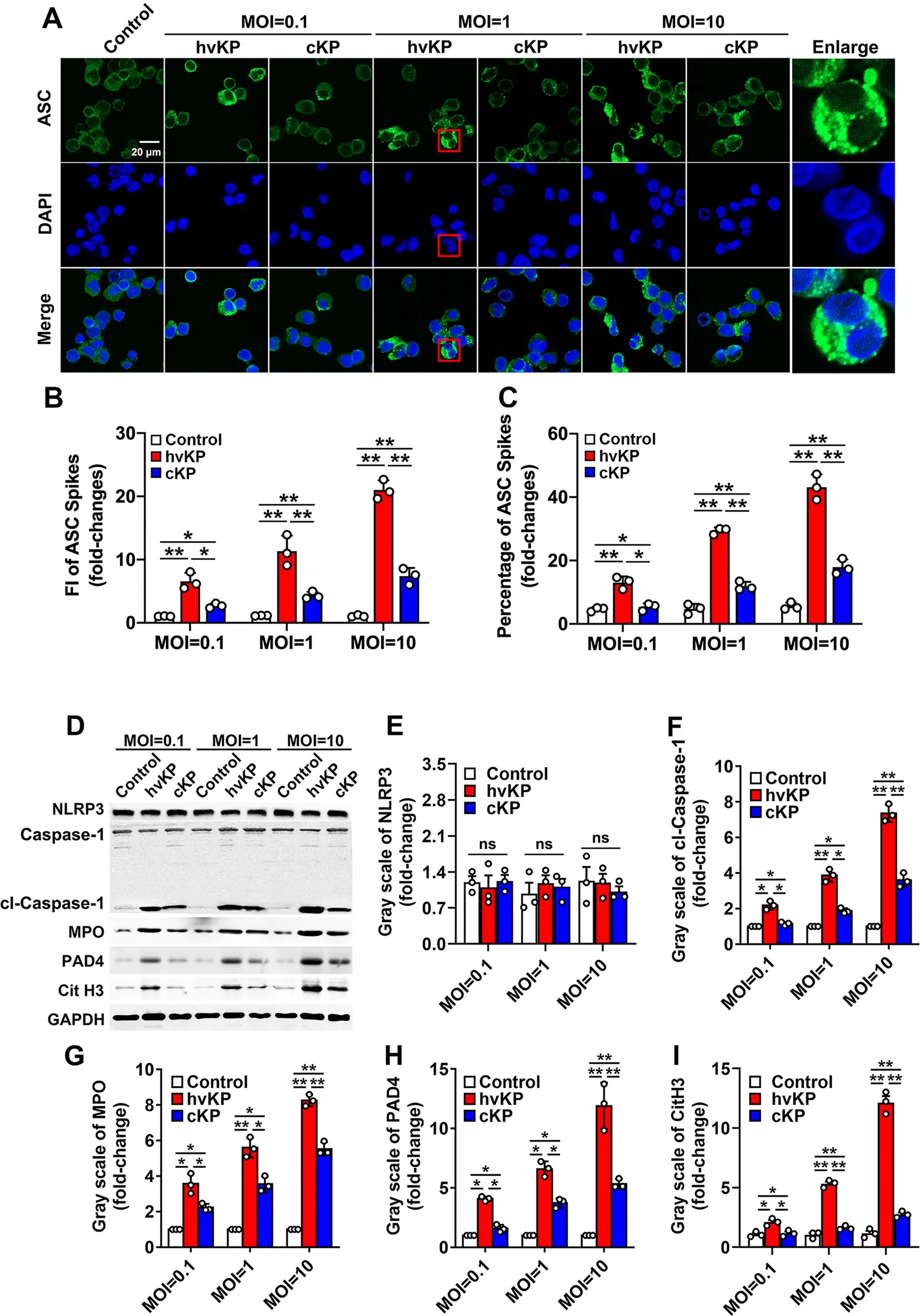
hvKP promotes NETosis. (**A**) Confocal microscopy to detect the influence of hvKP or cKP on ASC assembly. Scale bar = 20 µm. (**B**) FI of green fluorescence foci in experiments as described in panel A. The FI reflecting ASC foci in the control group was set as 1.0. (**C**) Percentage of ASC foci levels in total neutrophils. (**D**) The protein levels of NLRP3, Caspase-1, cl-Caspase-1, MPO, PAD4, and Cit H3 were detected by western blot. (**E – I**) Gray scale analyses of NLRP3, Caspase-1, cl-Caspase-1, MPO, PAD4, and Cit H3 immunoblotting bands in three experiments described in panel D. The gray scale values of immunoblotting bands from the untreated cells (control) were set as 1.0. Data are representative of three biological repeats in panels A–I (*n* = 3). *P < 0.05 and **P < 0.01.

**Fig 6:**
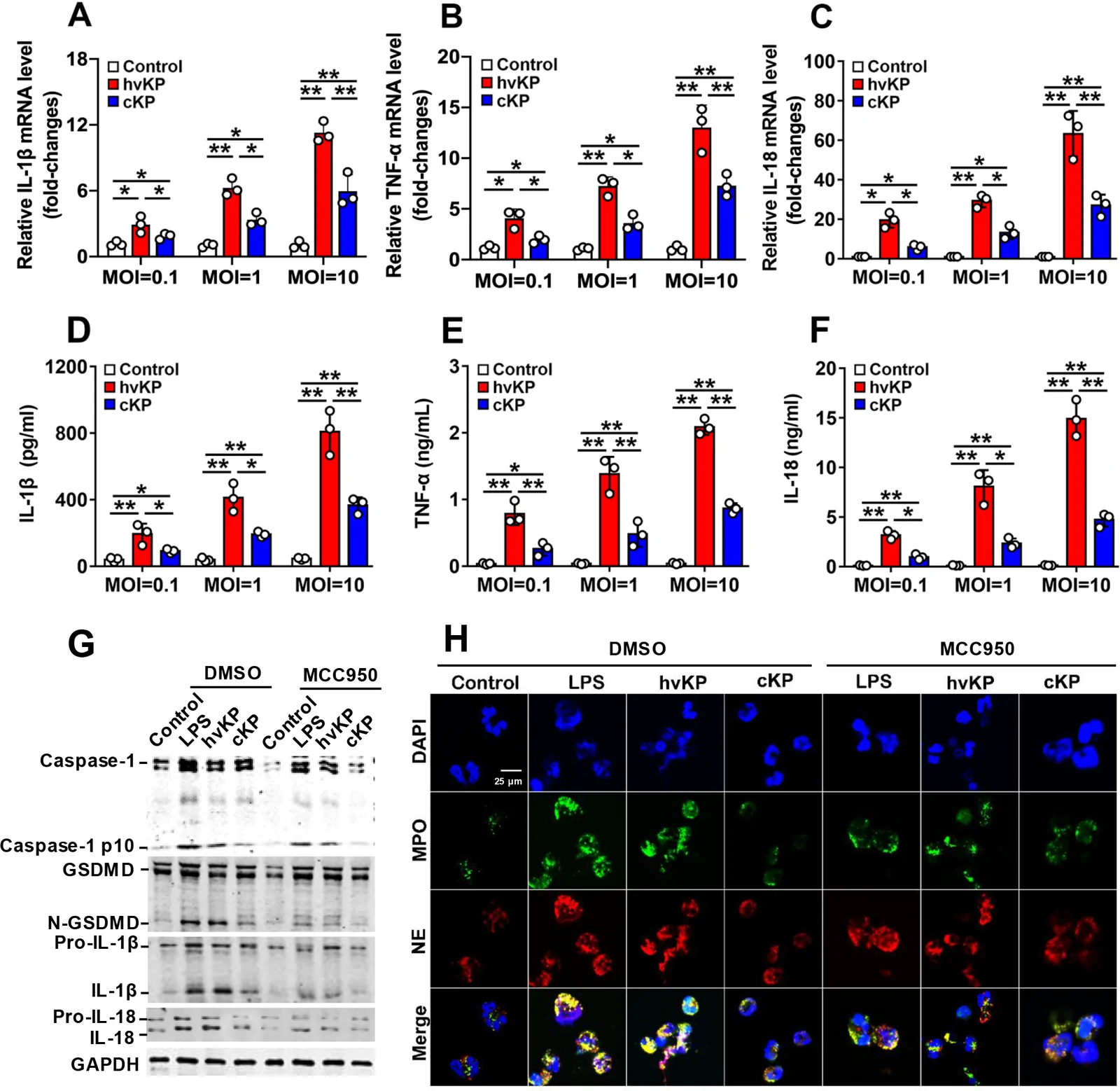
hvKP significantly promotes the activation of NLRP3 inflammasome and expression of NETosis-associated proteins in neutrophils. (**A–C**) mRNA levels of IL-1β, TNF-α, and IL-18 were detected by qRT-PCR, respectively. The mRNA level in the control group was set as 1.0. (**D–F**) Secretory levels of IL-1β, TNF-α, and IL-18 were detected by ELISA. (**G**) The protein levels of Caspase-1, cl-Caspase-1, GSDMD, N-GSDM, Pro-IL-1β, IL-1β, Pro-IL-18, and IL-18 in neutrophils infected with hvKP or cKP (with or without prior pretreatment with the NLRP3 inhibitor MCC950) were detected by western blot. (**H**) Fluorescence intensity of MPO and NE colocalization in neutrophils infected with hvKP or cKP (with or without prior pretreatment with the NLRP3 inhibitor MCC950) was detected by confocal microscopy. Scale bar = 25 µm. Mean ± SD of three independent experiments is shown. Data are representative of three biological repeats in panels **A–H** (*n* = 3). *P < 0.05 and **P < 0.01.

Inflammasomes play a pivotal role in the innate immune system, particularly in combating microbial infections (27). The NLRP3 inflammasome is triggered by various pathogens, yet excessive activation can lead to conditions that are linked to inflammation-associated diseases (28). NLRP3 and apoptosis-associated spotted protein (ASC) assemble into a large protein complex that forms foci, which serve as a marker for NLRP3 inflammasome activation (29). We observed the formation of ASC foci to check the influence of hvKP or cKP on neutrophil inflammasome activation. Our data showed that both hvKP and cKP promoted ASC assembly, but hvKP did so more significantly than cKP (Fig. 5A through C). The expression levels of NETosis-associated proteins Caspase-1, MPO, PAD4, and Cit H3, but not NLRP3, were significantly increased in neutrophils treated with hvKP compared to those treated with cKP (Fig. 5D through I), while mRNA levels of *PAD4* and *MPO* showed a similar trend, excluding *NLRP3* (Fig. S3A through C). To further investigate the production of inflammatory factors in neutrophils during infection by hvKP or cKP, mRNA and protein levels of cytokines were determined by qPCR and ELISA. Our data showed that both the transcription and expression levels of IL-1β, IL-18, and TNF-α were significantly increased in hvKP-infected neutrophils compared with those in cKP-infected neutrophils (Fig. 6A through F). Moreover, *in vitro* NLRP3 inhibition assay results demonstrated that in neutrophils pretreated with the NLRP3 inhibitor MCC950, the enhancing effects of hvKP or cKP on Caspase-1 and GSDMD cleavage, IL-1β and IL-18 expression, as well as neutrophil extracellular trap formation were markedly attenuated, compared with those in normally treated neutrophils (Fig. 6G and H). These data suggest that hvKP might promote NETosis by accelerating the activity of NLRP3 but not the expression in neutrophils.

### hvKP promotes neutrophil extracellular trap formation in a PAD4-dependent manner

To verify the association between hvKP-induced NET formation and PAD4, neutrophils were infected with hvKP or cKP (with or without prior pretreatment with the PAD4 inhibitor Cl-amidine). Western blotting and confocal microscopy revealed that in neutrophils pretreated with Cl-amidine, the enhancing effects of hvKP on the expression of PAD4 and Cit H3, as well as the co-localization of MPO and NE, were significantly decreased, compared with those in mice with normal infection (Fig. S3E and F). Furthermore, we observed partial co-localization of PAD4 and NLRP3 in the hvKP-infected group (Fig. S4). Our findings suggest that PAD4 is likely involved in hvKP-promoted NET formation in neutrophils.

### hvKP induces liver abscess by promoting NETosis formation through NLRP3 inflammasome activation *in vivo*

**Fig 7:**
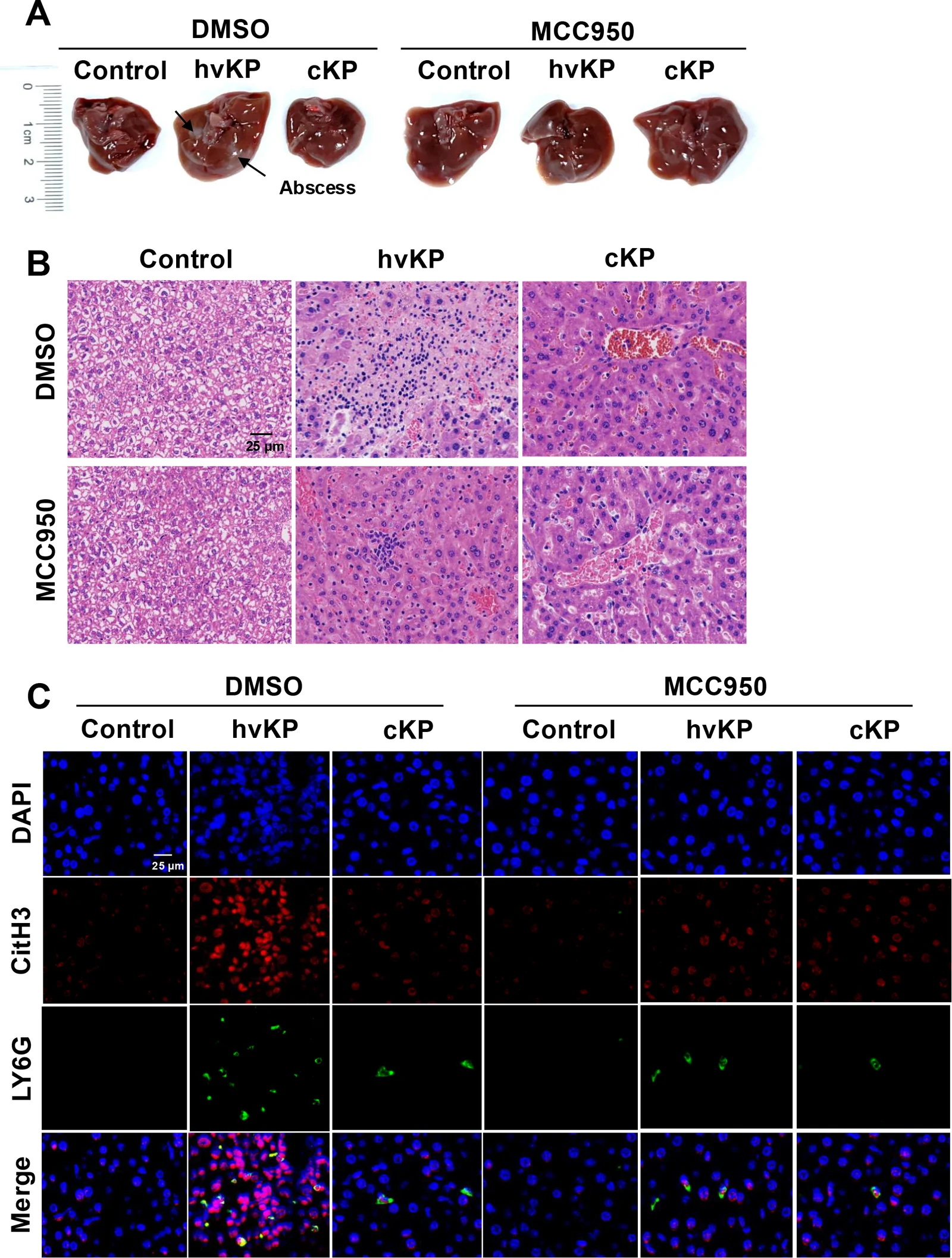
Pathogenicity of mice intraperitoneally infected with hvKP or cKP (with or without prior pretreatment with the NLRP3 inhibitor MCC950). (**A**) Gross pathological changes in livers from mice intraperitoneally infected with hvKP or cKP (with or without prior pretreatment with the NLRP3 inhibitor MCC950). (**B**) Histological examinations of livers from mice intraperitoneally infected with hvKP or cKP (with or without prior pretreatment with the NLRP3 inhibitor MCC950). Scale bar = 25 µm. (**C**) Level of Cit H3 in neutrophils within liver tissues *in situ* from mice intraperitoneally infected with hvKP or cKP (with or without prior pretreatment with the NLRP3 inhibitor MCC950) (*n* = 5). Scale bar = 25 µm. Data are representative of three biological repeats in panels A–C (*n* = 5).

To investigate the role of hvKP in inducing liver abscess by promoting NETosis formation through NLRP3 inflammasome activation *in vivo,* mice were intraperitoneally infected with hvKP or cKP (with or without prior pretreatment with the NLRP3 inhibitor MCC950). Results from the NLRP3 inhibition assay *in vivo* demonstrated that in mice pretreated with MCC950, the enhancing effects of hvKP on the induction of liver abscess formation, inflammatory cell infiltration in liver tissues, as well as the expression of Cit H3, were significantly attenuated compared with those in mice with normal infection (Fig. 7). This further confirms *in vivo* that hvKP induces liver abscess formation in mice through NLRP3 activation-mediated NET formation.

### The production of NETosis and cytokines was promoted in human peripheral blood samples from patients

**Fig 8:**
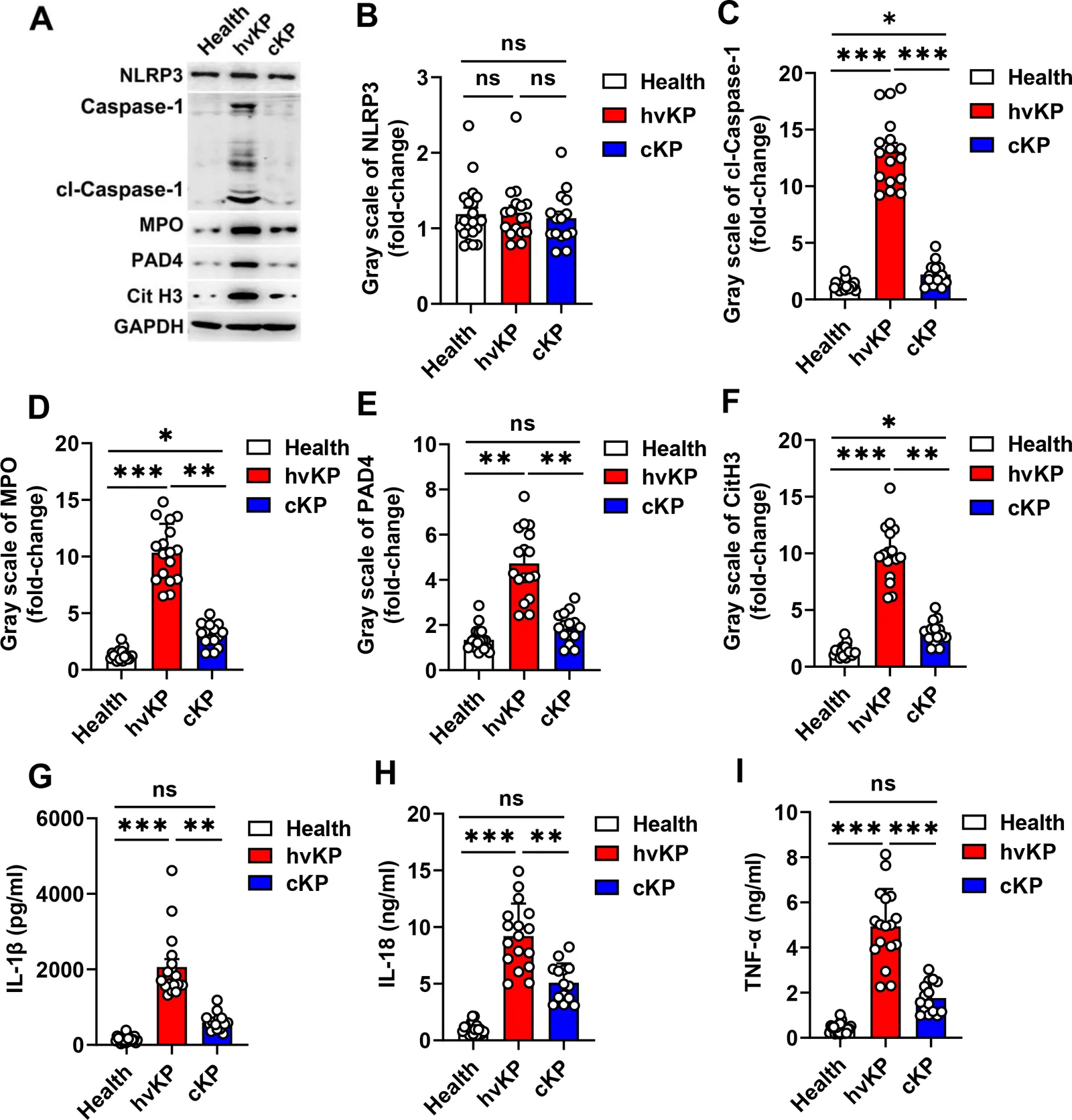
hvKP promotes NETosis-related protein expression and production of inflammatory cytokines in human peripheral blood samples from patients. (**A**) Western blot analyses of NLRP3, Caspase-1, cl-Caspase-1, MPO, PAD4, and Cit H3 expression in neutrophils of individuals without infection (Health) (*n* = 20), patients with hvKP infection (*n* = 17), and patients with cKP infection (*n* = 15). (**B–F**) Gray scale analyses of NLRP3, Caspase-1, cl-Caspase-1, MPO, PAD4, and Cit H3 immunoblotting bands in five experiments described in panel A. The gray scale values of immunoblotting bands from the untreated cells (control) were set as 1.0. (**G–I**) Secretory levels of IL-1β, TNF-α, and IL-18 in serum isolated from patients’ samples were detected by ELISA. *P < 0.05, **P < 0.01, and ***P < 0.001; ns, not significant.

We conducted a study to examine the protein levels of NETosis in neutrophils extracted from hvKP or cKP infections, as well as from healthy individuals. In samples from patients with hvKP, there was a notable increase in the levels of Caspase-1, MPO, PAD4, and Cit H3, but not in NLRP3, when compared to those with cKP (Fig. 8A through F). Similar elevations in the levels of Caspase-1, MPO, PAD4, and Cit H3 proteins were detected in mouse bone marrow neutrophils that were infected with hvKP (Fig. S4). Moreover, protein levels of IL-1β, IL-18, and TNF-α in patients’ serum were significantly higher in the hvKP-infected group compared to the cKP-infected group (Fig. 8G through I). In line with *in vitro* experiments, we successfully revealed that hvKP promoted more NETosis and cytokine production by activating NLRP3 than the cKP group.

## DISCUSSION

Liver abscess is a purulent hepatic infectious disease, with *Escherichia coli* and amoebae being the most common causative pathogens in the past (30, 31). Over the past few years, the incidence of liver abscess caused by *Klebsiella pneumoniae* infection in China, Asia, Europe, and the United States has been increasing, accounting for 43%–66% of the total incidence of bacterial liver abscess (32–36). It is even considered that *Klebsiella pneumoniae*-infected liver abscess is an invasive new infectious disease (37).

Hypervirulent *Klebsiella pneumoniae* has emerged as a clinically significant pathogen responsible for serious disseminated infections, including liver abscesses (8, 38, 39). It has been found that most of the *Klebsiella pneumoniae* causing liver abscesses have broad, thick capsules and high virulence, characteristics associated with hvKP rather than classical *Klebsiella pneumoniae* (40, 41). hvKP is generally associated with the hypermucoviscosity phenotype. An inoculation loop can generate a viscous string >5 mm in length from the bacterial colony, typically due to the increased production of capsular polysaccharide (CPS) and the presence of specific virulence genes, such as *magA*, *rcsA*, *rcsB, rmpA2,* and *rmpA* (42–45). *magA* (mucoviscosity-associated gene A) is essential for the synthesis of capsular polysaccharide and plays an important role in hypermucoviscosity, which is also linked to the pathogenesis of primary pyogenic liver abscess (45). In addition, high capsule productivity is also attributed to the regulation of the capsule synthesis genes (*rcsA* and *rcsB*), a regulator of mucoid phenotypes A and A2 (*rmpA* and *rmpA2*) (46–48). In the present study, we found that the hvKP but not cKP possess *magA*, *rcsA*, *rcsB, rmpA2,* and *rmpA* genes (Fig. S1A). In particular, hvKP has a viscous string about 58 mm in length observed visually compared with cKP, which was only 5 mm (Fig. S1B). Moreover, hvKP-infected mice exhibited more severe liver abscesses and higher mortality rates in (Fig. 1). In addition, hvKP exhibited greater survival within neutrophils (or exposure to preformed NETs) compared with cKP (Fig. S2D and S3D). Consistent with some reports, hvKP capsule genes serve as a central virulence node, with rmpA/rmpA2-mediated transcriptional activation driving the synthesis of a thick CPS (23). These hvKP-specific traits drive the paradoxical role of NETosis in liver damage: while NETosis acts as an innate defense to trap pathogens, hvKP virulence genes promote hypermucoviscous capsular synthesis, hyperactivating neutrophils to trigger excessive, dysregulated NET release. Cytotoxic NET components directly damage hepatocytes, induce NLRP3-mediated pyroptosis, and amplify inflammation. hvKP may further evade NET killing via DNase secretion, fueling a vicious cycle of non-functional NET accumulation, persistent infection, and tissue destruction, ultimately exacerbating the pathological outcomes observed in our mouse model. Our findings demonstrate that the synthesis and regulation of CPS genes are critical for contributing to NETosis formation for tissue injury rather than bacterial clearance.

Reactive oxygen species play a pivotal role in cell signaling by regulating cell proliferation and survival (49, 50). Infections, inflammatory cytokines, and other environmental cues can raise ROS levels to detrimental concentrations that contribute to pathological disease manifestation, which is linked to cell death or mediates (51, 52). We observed significantly increased numbers of intracellular viable bacteria and chromatin depolymerization and ROS levels in hvKP-treated neutrophils compared with the cKP treatment group (Fig. 2; Fig. S2). These data imply that thick capsules of hvKP not only subvert the innate immunity but also enhance the pathogenicity in neutrophils.

PAD4 is an enzyme that is expressed in neutrophils, which can drive the formation of neutrophil extracellular traps (13, 18, 53). NLRP3 inflammasome assembly in neutrophils is supported by PAD4 and promotes NETs (54). NETs possess antibacterial activity, which is ascribed to the associated histones, proteolytic enzymes from granules that might degrade bacterial virulence factors, and enzymatically active myeloperoxidase (12, 55). It was reported that the pro-inflammatory role of NETs was documented in a mouse model of ischemia-reperfusion injury, in which NETs amplify inflammation and liver damage (56, 57).

Our transcript data show that levels of PAD4 and MPO were significantly upregulated in hvKP-infected neutrophils compared to the cKP-treated group (Fig. 4D). Interestingly, the hvKP-infected group promoted the nod-like receptor signaling pathway in neutrophils when compared with the cKP-infected group (Fig. 4A) but did not affect transcription (Fig. 4E; Fig. S3A) and expression (Fig. 5D, E, 8A, and B) levels of NLRP3. NLRP3 activation adheres to a two-step priming-activation model: priming upregulates NLRP3 transcription via NF-κB and posttranslationally modifies NLRP3 to an autoinhibited pre-activated state, while activation is induced by diverse stimuli converging on K^+^ efflux or other ionic perturbations (58). It has been reported that the capsule of hvKP can impede phagosome-lysosome fusion, leading to increased lysosomal membrane permeability and cathepsin B release, which, in turn, activates NLRP3 via a pathway independent of NF-κB signaling (59). Therefore, this may explain why hvKP promotes NETosis via NLRP3, yet no increase in NLRP3 expression is observed.

It was reported that NET formation promotes the secretion of pro-inflammatory cytokines in neutrophils (13, 25). To compare hvKP with cKP in the efficiency to enhance the expression levels of cytokines, human neutrophils were treated with hvKP and cKP, respectively. Although both hvKP and cKP significantly enhance mRNA transcription and secretion of IL-1β, TNF-α, and IL-18 compared to the control group, hvKP is more efficient than cKP (Fig. 6 and 7G through I). These data, together with the promoting effect of ASC assembly (Fig. 5A through C), imply that hvKP is more potent than cKP in accelerating the activation of NLRP3 and the production of NETosis.

**Fig 9:**
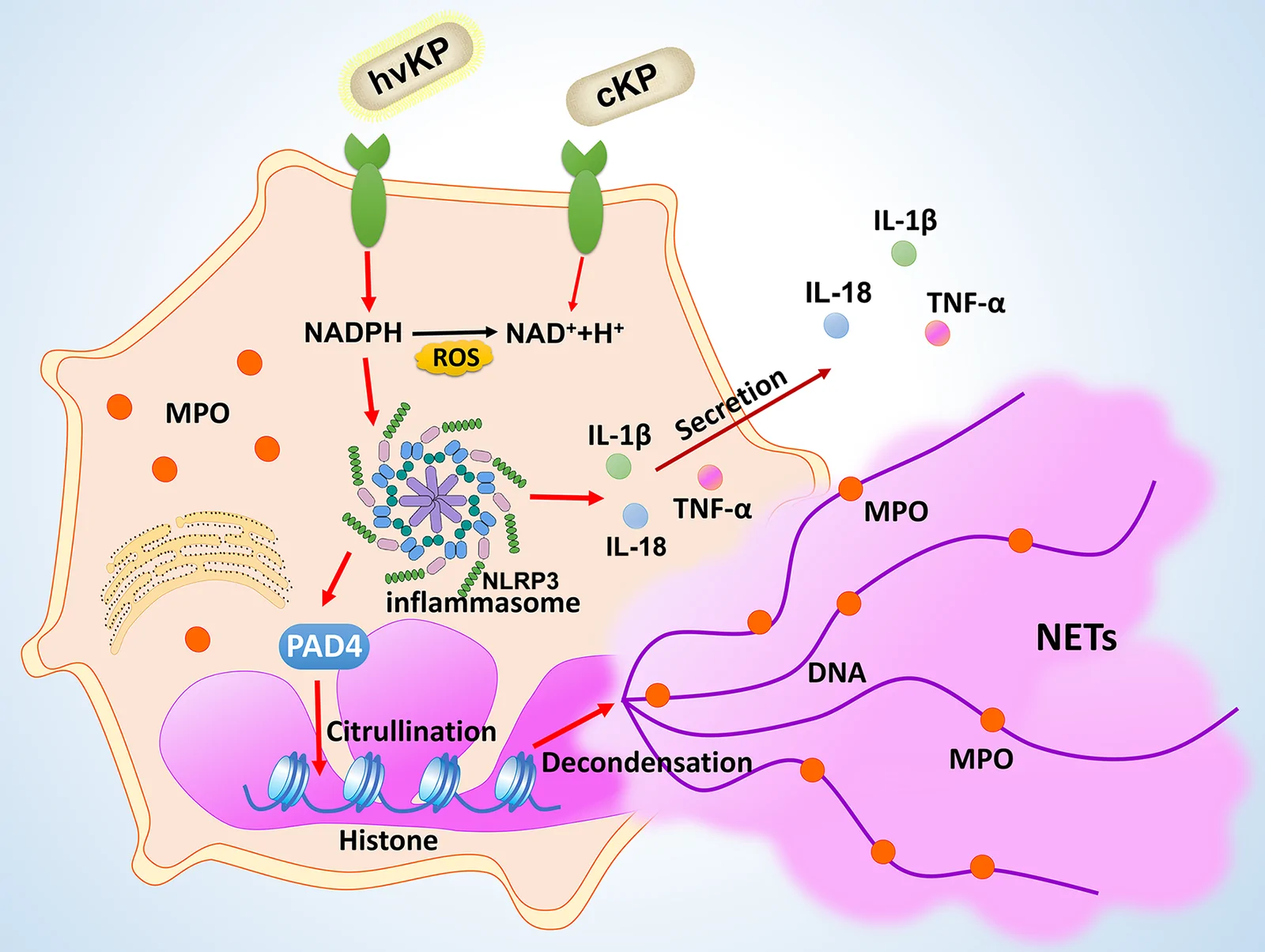
Schematic diagram of the mechanisms underlying how hvKP promotes neutrophil extracellular trap formation by activating the NLRP3 inflammasome. Upon hvKP infection, NADPH oxidase catalyzes the conversion of intracellular O_2_ into ROS. ROS activate the NLRP3 inflammasome to secrete IL-1β, TNF-α, and IL-18. The NLRP3 inflammasome activates downstream NETosis essential enzymes, such as MPO and PAD4. NETs digest nucleosomal histones and promote chromatin relaxation, which favors PAD4 to catalyze histones into citrullinated histones. Citrullination of histones causes chromatin entropic swelling and release of NETs.

Altogether, the above results indicate that hvKP promotes neutrophil extracellular trap formation by activating the NLRP3 inflammasome (Fig. 9). However, these results collectively emphasize the hvKP infection. Further research is required to investigate which virulence factor of hvKP is responsible for the activation of NLRP3 and the induction of NETosis in neutrophils.

In our *in vitro* and *in vivo* experiments, we relied on one hvKP strain and one cKP strain to investigate bacterial-host interactions. This limitation restricts the generalizability of our mechanistic findings, as *K. pneumoniae* strains exhibit high genetic and phenotypic diversity. The sample size of our patient cohort (hvKP-infected patients and cKP-infected patients after propensity score matching) is relatively small, which may limit the statistical power of our analyses. Future studies will include a panel of clinical isolates (at least 5–10 hvKP and 5–10 cKP strains) with diverse genetic backgrounds and phenotypic characteristics. We will use isogenic strains to further validate the specific effects of hypervirulence factors on host responses. We will also expand the cohort by collaborating with multiple hospitals to enroll a larger, more diverse population of *K. pneumoniae*-infected patients, including those from different geographic regions and with varying clinical backgrounds. This will enable us to validate our current findings and explore additional associations that may have been missed due to the limited sample size.

## MATERIALS AND METHODS

### Bacterial strains and culture

*Klebsiella pneumoniae* strain NTUH-K2044 (GenBank accession no.: NC_012731) (hvKP) and classic *Klebsiella pneumoniae* strain HS11286 (GenBank accession no.: NC_016845) (cKP) were cultured in a blood agar plate and LB medium at 37°C.

### Mouse liver abscess model

Six- to eight-week-old pathogen-free female C57BL/6 mice were provided by Slack Laboratory Animals Co., Ltd (Shanghai, China). Mice were intraperitoneally inoculated with 10^8^ colony-forming units (CFUs) of hvKP or cKP. Normal saline-treated mice served as the control group. The model mice were sacrificed, and the livers were obtained for pathological examination after 72 h (60).

### Histological examination

The livers from murine models were fixed in 4% paraformaldehyde (PFA) for 24 h at room temperature. After fixation, the tissue was immersed in different concentrations of ethanol for dehydration and then cleared with xylene before being embedded in paraffin wax. The embedded tissues were cut into sections and stained with htx-eosin as described previously (60). Finally, the histopathological changes were observed by microscope.

### Virulence testing in the mouse infection model

Overnight cultures of hvKP and cKP were diluted in sterile phosphate-buffered saline to obtain a concentration of 10^8^ CFU/mL. Six-week-old pathogen-free female C57BL/6 mice (five per group) were inoculated intraperitoneally with hvKP or cKP that had been washed twice with normal saline (23). The survival rate of hvKP or cKP was recorded at 1, 2, 3, 4, and 5 days.

### Detection of virulence genes in hvKP and cKP

Genomic DNAs from the hvKP and cKP strains were extracted using a Bacterial Genomic DNA Extraction Kit (Axygen, USA). The entire *wzy-K1*, *rcsA*, *rcsB*, *rmpA*, and *rmpA2* genes were amplified from the DNA templates by PCR with corresponding primers (Table S1) using a High-Fidelity PCR Kit (TaKaRa, Japan). All the PCR products were examined by 1.5% ethidium bromide pre-stained agarose gel electrophoresis.

### String test

String tests were performed thrice per strain as described previously (61). hvKP and cKP were cultured on blood agar plates (Bio-Kont, China) and incubated overnight at 37°C. An inoculating loop was used to touch the colonies gently and then lifted. The test was considered positive when the produced string was longer than 5 mm.

### Isolation and purity detection of human peripheral blood neutrophils

Human peripheral blood neutrophils were isolated using EasySep Direct Human Neutrophil Isolation Kit (Stemcell Technologies, USA) as described previously (26). Subsequently, the purity of the human neutrophils was detected by fluorescence-activated cell sorting (FACS) using antibodies against CD11b and CD66b (Thermo Fisher, USA).

### Detection of viability of hvKP and cKP from infected neutrophils

Neutrophils (1 × 10^6^ cells) were co-cultured with hvKP or cKP at MOIs of 0.1, 1, and 10 for 4 h. After washing with PBS and trypsinization, the co-cultured cells were collected and centrifuged at 300 × *g* at 4°C for 5 min. After incubation with 100 U/mL penicillin and 100 μg/mL streptomycin for 30 min, the cells were washed with PBS three times to remove extracellular bacteria in the supernatants and then lysed with 0.05% NaTDC-PBS to release intracellular bacteria. The lysates were centrifuged at 12,000 × *g* at 4°C for 10 min to precipitate intracellular bacteria. In the DNase assay, 1 × 10^6^ cells of human neutrophils after exposure to pre-formed NETs (treated with or without DNase I [Selleckchem, USA]) were co-cultured with hvKP or cKP at an MOI of 1 for 4 h. Subsequently, hvKP or cKP counts were determined over time by colony-forming units per mL by spot-plating serial dilutions on blood agar plates protected by a gentamicin (50 μg/mL) (MCE, USA) coating in triplicate. Additionally, the bacteria were stained by LIVE/DEAD Bacterial Viability Kit (Thermo), and the viability of bacteria was observed by confocal microscopy (Olympus, Japan) (485/630 nm excitation/emission wavelengths for SYTO 9) as previously described (60, 62). hvKP and cKP, which were cultured in LB medium, were used as the control groups.

### Confocal microscopy to detect ROS production

Neutrophils (1 × 10^6^/mL) were co-cultured with different MOIs (0.1, 1, and 10) of hvKP or cKP for 240 min, and untreated cells served as the control group. Cells were resuspended in 1 mL of serum-free culture medium and stained with 1 μL of DCFH-DA probe dye (Invitrogen, USA) in the dark for 20 min at 37°C. After washing three times with PBS, cells in each group were adjusted to a density of 1 × 10^6^ cells/mL. The ROS of neutrophils was detected by flow cytometry (BD, USA) and observed by confocal microscope (Olympus, Japan) (26). The data were analyzed by FlowJo, and confocal images were measured with the ImageJ software.

### Confocal microscopy to observe NETosis

The above-treated neutrophils and neutrophils infected with hvKP or cKP (with or without prior pretreatment with the MCC950 or Cl-amidine) were fixed by 4% PFA at room temperature and then permeabilized with 0.1% Triton-X-100 in PBS for 20 min. After washing three times with PBS, cells were blocked with 1% BSA and 0.1% Tween 20 in PBS for 2 h at room temperature. Cells were incubated with rabbit anti-MPO antibodies, anti-NE antibodies, anti-PAD4 antibodies, or anti-NLRP3 antibodies (Abcam, UK) overnight at 4°C. Neutrophils were incubated in the dark with goat anti-rabbit IgG antibody (Alexa Fluor 594 nm, 1:500 diluted in PBS) for 2 h at room temperature and stained with 100 ng/mL DAPI for 30 min at room temperature. Following staining, cells were washed, and ProLong Diamond Antifade Mountant (Thermo, USA) was added to each coverslip. The coverslips were placed onto the slides and then observed with a confocal fluorescence microscope (Olympus, Japan). Images were analyzed with the ImageJ software as previously described.

### Quantification of NETs

NETosis was quantified by detecting DNA release with the DNA-binding dye Sytox Green (26). Neutrophils were co-cultured with hvKP or cKP (MOI = 10) for 240 min, and then the cells were harvested to detect DNA release by staining with Sytox Green (50 nM) every 30 min during co-culture. Fluorescence was measured with 488 nm excitation, and the scale was set and threshold adjusted to define the area of fluorescent DNA. The total area of all fluorescent particles indicated the amount of NETosis. Neutrophil extracellular trap area and colocalization of NET markers were quantified using ImageJ (https://fiji.sc) from 8-bit fluorescence images containing a NET-specific channel, a colocalization marker channel, and an optional DAPI channel. For NET area quantification, images were imported and split. A Gaussian blur was optionally applied to reduce noise. Consistent thresholds (default for 8-bit, adjusted via negative controls) were applied, followed by conversion to a binary mask. Small artifacts were removed via watershed and particle analysis, and total NET area was calculated by summing the segmented regions. For colocalization quantification, the two channels were split and preprocessed consistently, and alignment was performed if necessary. The same thresholds used for individual segmentation were applied, and Pearson’s correlation coefficient (PCC) was obtained, ensuring the standardized pipeline guarantees accurate and reproducible results. Unstimulated neutrophils had an area of 62 ± 6.1 µm^2^. Thus, only particles >70 µm^2^ were considered NETs.

### ASC foci imaging

Treated neutrophils were permeabilized with 0.1% Triton-X-100, blocked with 1% BSA as previously described, and then fixed with 4% PFA. Then, the cells were incubated with rabbit anti-TMS1/ASC antibodies (Abcam, UK) overnight at 4°C, washed three times, and incubated with Alexa Fluor 488 nm goat anti-rabbit IgG fluorescent secondary antibody. After washing with PBS, ProLong Diamond Antifade Mountant (Thermo, USA) was added to prevent fluorescence quenching. Aggregation of ASC was observed by a confocal fluorescence microscope (Olympus, Japan).

### Transcriptome sequencing

Neutrophils (1 × 10^6^ cells) were infected for 120 min with hvKP and cKP, respectively, and untreated neutrophils served as the control group. Cells were collected (1,000 × *g* for 10 min) and frozen at −80°C with RNAiso Plus (TaKaRa, Japan). Total RNA was extracted, and a genome-wide transcriptomics analysis was conducted (LC-Bio Technologies Hangzhou Co., Ltd.). For quality control (QC) metrics, raw reads were filtered to remove adapter sequences, low-quality reads (Q-value < 20), and reads containing >10% unknown nucleotides. Clean reads were evaluated for GC content, error rate, and mapping efficiency against the reference genome using HISAT2. Key QC parameters, including clean read yield (>6 Gb per sample), Q30 score (>90%), and genome mapping rate (>85%), were met for all samples. Replicate concordance was assessed by calculating PCCs of gene expression levels (FPKM/TPM values) between biological replicates using R software. High correlation coefficients (PCC > 0.95) were observed across all replicate groups, confirming high reproducibility of the RNA-seq data. The biological functions and signaling pathways of differentially expressed genes (DEGs) identified from RNA-Seq data were analyzed through enrichment analysis using Gene Ontology (GO) databases, Fisher’s exact test, and Gene Set Enrichment Analysis (GSEA); Fisher’s exact test was used to determine the significant enrichment of DEGs in specific GO term pathways (*P* < 0.05 as the threshold), while GSEA evaluated the enrichment of pre-defined gene sets from GO databases in the entire ranked gene list based on expression fold changes (false discovery rate < 0.25 and nominal *P* < 0.05 as significant thresholds), and all analyses were implemented using the clusterProfiler R package in R software. The differentially expressed mRNAs were selected with fold change >2 or fold change <0.5 and *P*-value < 0.05 by R package DESeq2 (http://www.bioconductor.org/packages/release/bioc/html/DESeq2.html).

### qPCR

To detect the influence of hvKP or cKP on mRNA expression of *IL-1β*, *TNF-α*, *IL-18*, *NLRP3*, *PAD4,* and *MPO,* total RNA was extracted from neutrophils co-cultured with hvKP or cKP (MOI = 0.1, 1, and 10) as described above. RNAs were reverse transcribed and used for qRT-PCR to detect the expression levels of *IL-1β*, *TNF-α*, and *IL-18*. Data were analyzed using the ΔΔCt model and randomization test in REST 2005 software (63, 64). The primers used in qRT-PCR are listed in Table S1.

### *In vitro* inhibition assay of PAD4

To determine whether the formation of extracellular traps on PAD4 and the position of PAD4 relative to NLRP3 in neutrophils exposed to hvKP depend on it, 1 × 10^6^ cells per well of human peripheral blood neutrophils were cultured in 6-well culture plates (Corning, USA) overnight. Then, the cells were infected with hvKP or cKP (MOI = 1) for 4 h or pretreated with 5 μM of Cl-amidine (Selleckchem, USA) for 30 min, respectively. The expression of PAD4 and Cit H3 and co-localization of MPO and NE were detected by western blot and confocal microscopy, respectively.

### *In vivo* inhibition assay of NLRP3

Six- to eight-week-old pathogen-free female C57BL/6 mice were intraperitoneally infected with 10^8^ CFU of hvKP or cKP (with or without prior pretreatment with the NLRP3 inhibitor MCC950) (Selleckchem, USA, 10 mg/kg of body weight). Subsequently, the gross appearance and histological lesions of the liver were observed by hematoxylin-eosin staining, and the expression level of Cit H3 (Abcam, UK) in neutrophils from mouse liver tissues was detected by immunofluorescence after 72 h.

### Human peripheral blood samples infected with *Klebsiella pneumoniae*

A total of 32 peripheral blood samples were collected from patients infected with hvKP and cKP hospitalized in the Second Affiliated Hospital of Zhejiang Chinese Medical University and 20 healthy volunteers (male = 10 and female = 10), with ages ranging from 35 to 72 (46.62 ± 8.44) as a control group. There is no potential bias for recruiting the patient cohort. A total of 32 consecutive patients with *K. pneumoniae* infection were enrolled in this study, among whom 17 were confirmed as hvKP infected (male = 12 and female = 5), with ages ranging from 47 to 75 (49.36 ± 10.08), and 15 as cKP infected (male = 9 and female = 6), with ages ranging from 43 to 68 (42.11 ± 7.99), based on the positive detection of hypervirulence-associated genes (*rmpA*, *rmpA2*, and *magA*) and string test assays. No significant difference in age or gender was observed between the two groups (*P* > 0.05), confirming the balance of baseline demographic characteristics. The most common comorbidities in the hvKP group included diabetes mellitus, hypertension, and chronic liver disease, while those in the cKP group included diabetes mellitus, hypertension, and chronic kidney disease. There are no statistically significant differences in the overall comorbidity burden between the two groups (*P* > 0.05). The primary infection sites for hvKP were pyogenic liver abscess, pneumonia, and bloodstream infection, while those for cKP were urinary tract infection, pneumonia, and bloodstream infection. For all patients, clinical samples for *K. pneumoniae* isolation were collected within 24 h of initial diagnosis of infection and prior to the administration of any empirical or targeted antibiotics. For disease stage stratification, sampling was performed at the acute phase of infection (defined as <72 h after the onset of symptoms) for all enrolled patients, ensuring consistency in disease severity at the time of sample collection. To clarify the critical point of serum collection, serum samples were collected from all patients at a comparable time point: within 24–48 h after the confirmation of *K. pneumoniae* infection and prior to antibiotic administration. This ensures that serum biomarkers were measured under consistent clinical conditions, eliminating potential biases caused by differential timing of sample collection between the two groups. Blood cultures were used to confirm *Klebsiella pneumoniae* infection, as well as protein-purified derivative and serology tests at the same time to exclude infections by other common pathogens. No other pathogens were found by these tests. Peripheral blood samples were obtained from the patients on admission. Informed consent was waived as this study used leftover clinical samples (residual blood specimens from routine diagnostic tests) that were de-identified and would otherwise have been discarded. All methods complied with the Declaration of Helsinki.

### ELISA

The expression of IL-1β, TNF-α, and IL-18 in the supernatants of neutrophils with different treatments and in the serum of blood samples from the patients was measured by ELISA according to the manufacturer’s instructions (Novus, USA).

### Western blot

Sediments from treated cells were harvested, and neutrophils were isolated from the blood of patients. Cells were then lysed using RIPA lysis buffer. Total protein from the lysate was collected by centrifugation at 4°C at 12,000 × *g*. To detect the expression levels of NETosis-associated proteins and NLRP3, samples from each group were subjected to SDS-PAGE and electrotransferred onto PVDF membrane (Bio-Rad, USA). NLRP3, Caspase-1, GSDMD, MPO, PAD4, Cit H3, pro-IL-1β, IL-1β, pro-IL-18, and IL-18 were probed using rabbit anti-NLRP3-IgG, rabbit anti-Caspase-1-IgG, rabbit anti-GSDMD-IgG, rabbit anti-MPO-IgG, rabbit anti-PAD4-IgG, rabbit anti-Cit H3-IgG (Abcam, UK), rabbit anti-pro-IL-1β, rabbit anti-IL-1β, rabbit anti-pro-IL-18, and rabbit anti-IL-18 (ABclonal, China) as the primary antibodies, respectively (65, 66). IRDye 680RD goat anti-rabbit-IgG (H + L) (LI-COR, USA) was used as the secondary antibody. The images were developed on the ODYSSEY CLx Infrared Imaging System (LI-COR). Gray scale of bands was analyzed by ImageJ, and each group had at least three independent experiments. GAPDH was used as the internal reference.

### Statistics analyses

Data shown are mean ± SD of three independent experiments. Images were analyzed by ImageJ software. Dunnett and Mann-Whitney tests in SPSS 22.0 were used to determine significant differences. Data from quantification of NET formation were analyzed by Mann-Whitney test as previously described. Data from survival was analyzed by Kaplan-Meier with a log-rank test. Multiple-group/time-course data were assessed by two-way analysis of variance with appropriate *post hoc* correction. *P* < 0.05 is considered to be statistically significant, and *P* < 0.01 is extremely significant. NS is considered to be not significant. All source data are provided in a separate raw data file of supplemental material.

## SUPPLEMENTAL MATERIAL

Fig. S1 spectrum.02376-25-s0001.tif Virulence gene and mucoviscosity phenotype in hvKP and cKP.

Fig. S2 spectrum.02376-25-s0002.tif Intracellular survival of hvKP in neutrophils.

Fig. S3 spectrum.02376-25-s0003.tif hvKP promotes NETs formation via PAD4.

Fig. S4 spectrum.02376-25-s0004.tif Partial co-localization of PAD4 and NLRP3 in hvKP-infected neutrophils.

Fig. S5 spectrum.02376-25-s0005.tif hvKP promotes the NETosis related proteins levels in neutrophils from mouse bone marrow.

Table S1 spectrum.02376-25-s0006.docx Primers used in this study.

ASM does not own the copyrights to Supplemental Material that may be linked to, or accessed through, an article. The authors have granted ASM a non-exclusive, world-wide license to publish the Supplemental Material files. Please contact the corresponding author directly for reuse.
