# Supplementary figures and images for: Hypervirulent Klebsiella pneumoniae induces liver abscess by promoting neutrophil extracellular trap formation through NLRP3 inflammasome activation
Source: Microbiol Spectr. 2026 May 18;14(7):e02376-25. doi: 10.1128/spectrum.02376-25 (PMC13340210; doi:10.1128/spectrum.02376-25)

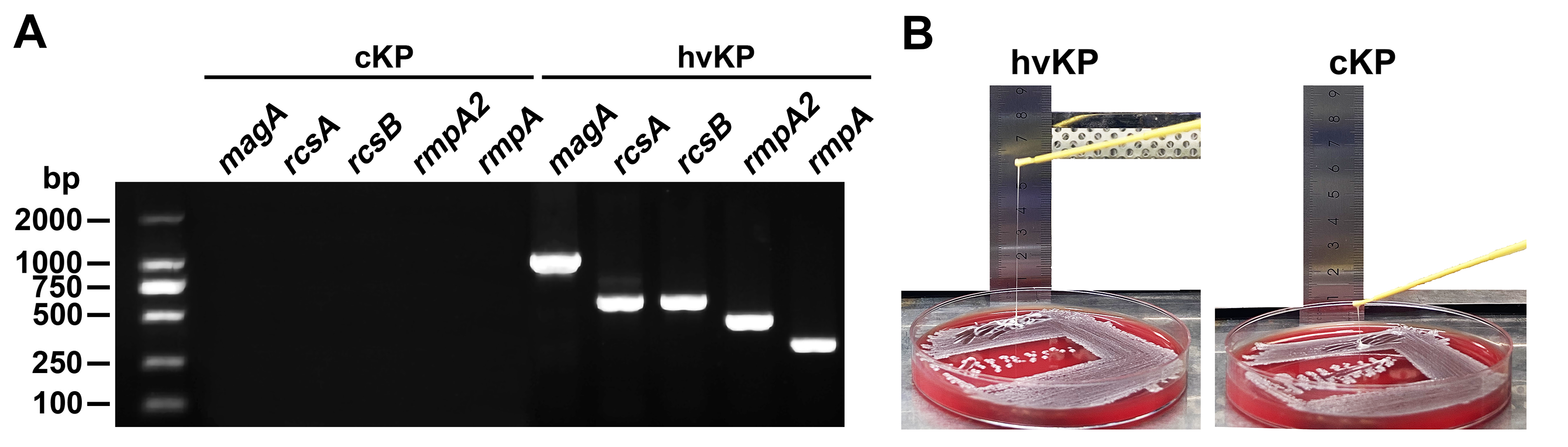

Supplement: Fig. S1 — Virulence gene and mucoviscosity phenotype in hvKP and cKP. [file spectrum.02376-25-s0001.tif]

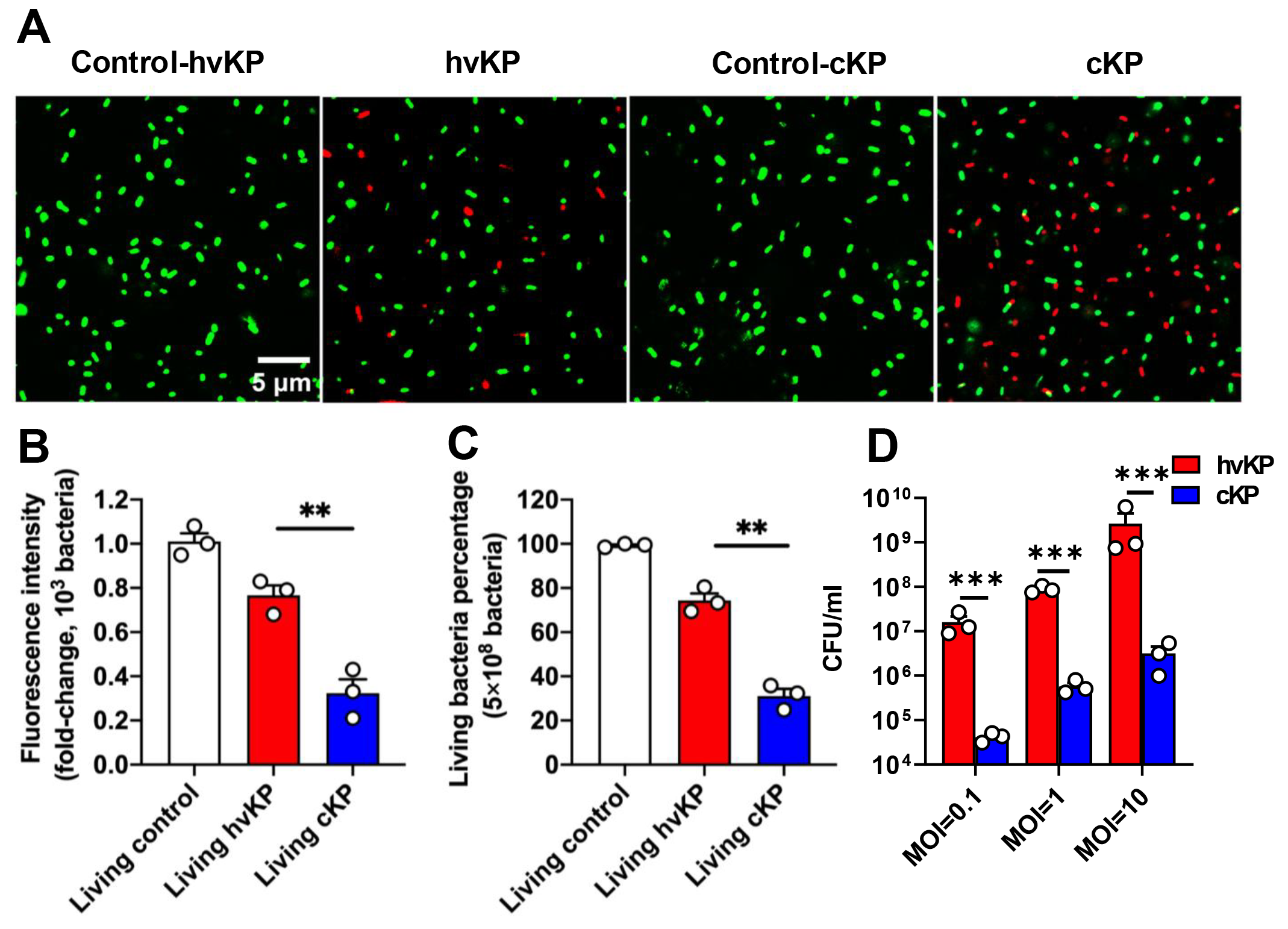

Supplement: Fig. S2 — Intracellular survival of hvKP in neutrophils. [file spectrum.02376-25-s0002.tif]

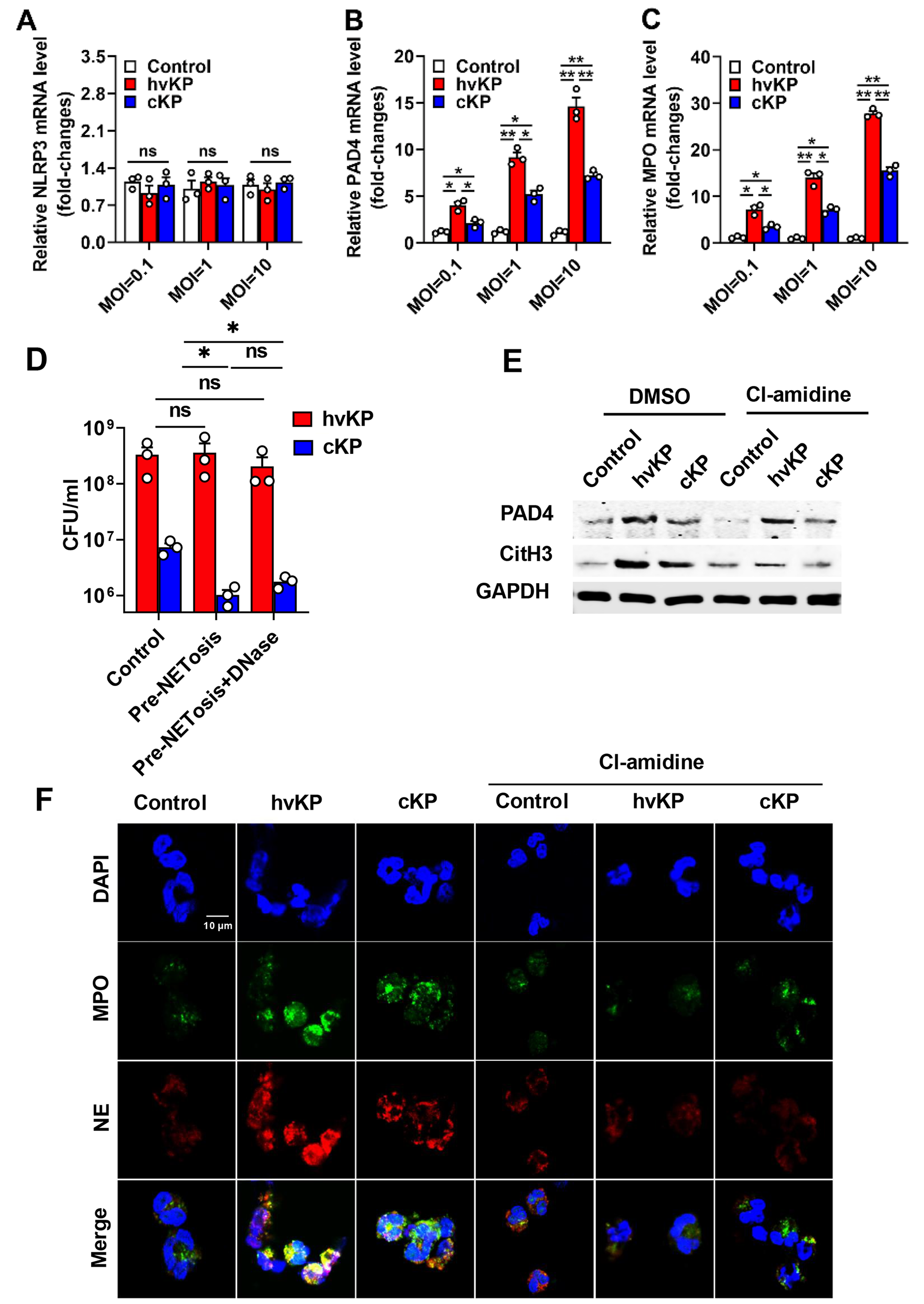

Supplement: Fig. S3 — hvKP promotes NETs formation via PAD4. [file spectrum.02376-25-s0003.tif]

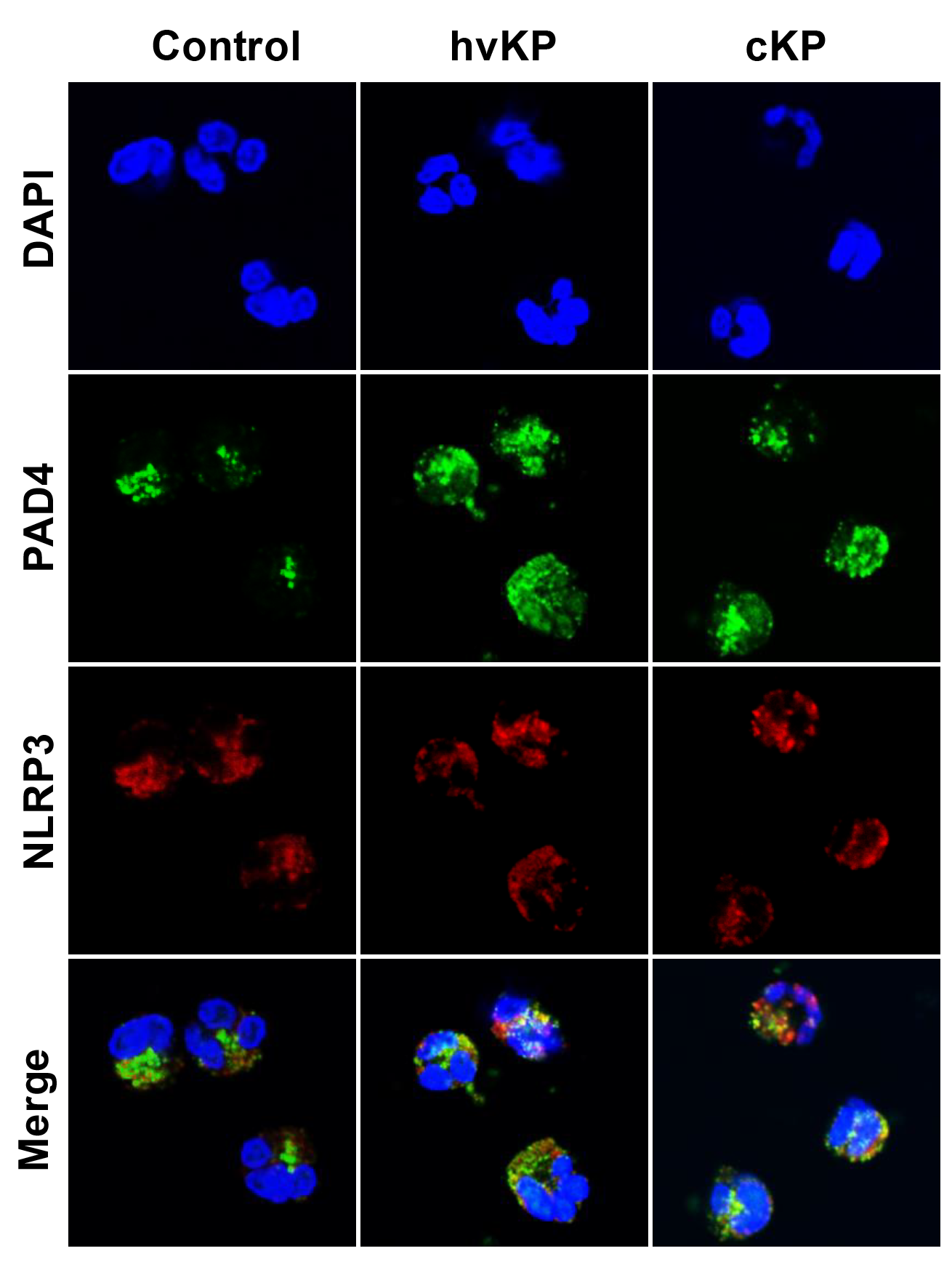

Supplement: Fig. S4 — Partial co-localization of PAD4 and NLRP3 in hvKP-infected neutrophils. [file spectrum.02376-25-s0004.tif]

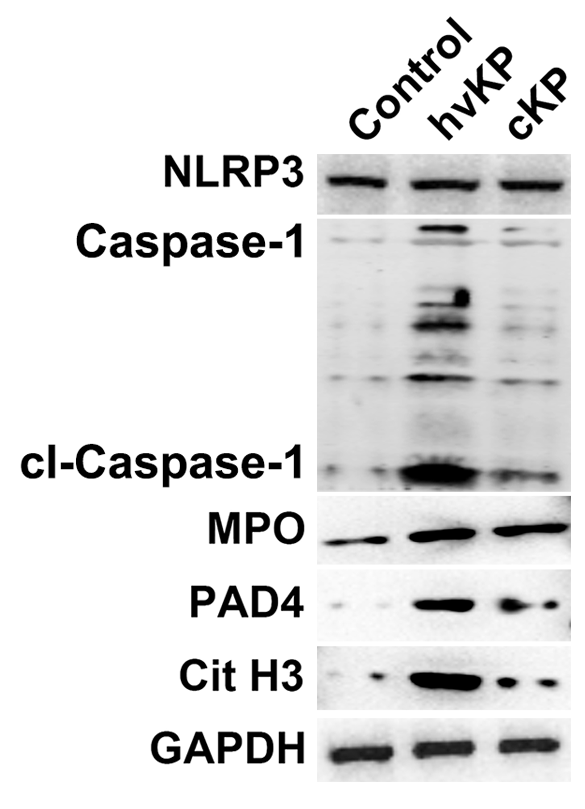

Supplement: Fig. S5 — hvKP promotes the NETosis related proteins levels in neutrophils from mouse bone marrow. [file spectrum.02376-25-s0005.tif]
