# Supplementary material for: Hypervirulent Klebsiella pneumoniae induces liver abscess by promoting neutrophil extracellular trap formation through NLRP3 inflammasome activation
Source: Microbiol Spectr. 2026 May 18;14(7):e02376-25. doi: 10.1128/spectrum.02376-25 (PMC13340210; doi:10.1128/spectrum.02376-25)
Supplement: Table S1 — Primers used in this study. [file spectrum.02376-25-s0006.docx]

Table S1 Information of primers used in this study

| Primer | Sequence (5’ to 3’) | Purpose |
| --- | --- | --- |
| *magA* | F: GGTGCTCTTTACATCATTGC | Detection of emtire *magA* gene |
|  | R: GCAATGGCCATTTGCGTTAG |  |
| *rcsA* | F: ATGTAAACGATGATTATGGATT | Detection of entire *rcsA* gene |
|  | R: TCAGCGCATATTTACCTGAA |  |
| *rcsB* | F: ATGAACACTATGAACGTAATTATT | Detection of entire *rcsB* gene |
|  | R: TTACTCTTTGTCCGTCGC |  |
| *rmpA2* | F: GTGCAATAAGGATGTTACATTA | Detection of entire *rmpA2* gene |
|  | R: GACTTATCATATTTAATGTT |  |
| *rmpA* | F: ATGGAAAAATATTTACTTTAT | Detection of entire *rmpA* gene |
|  | R: CTAAATACTTGGCATGAGCCA |  |
| *il-1β* | F: CCTTGTGCAAGTGTCTGAAG | Detection of *il-1β*-mRNA  Detection of *tnf-α*-mRNA  Detection of *il-18*-mRNA  Detection of *nlrp3*-mRNA  Detection of *pad4*-mRNA  Detection of *mpo*-mRNA |
| *tnf-α*  *il-18*  *nlrp3*  *pad4*  *mpo* | R: GGGCTTGGAAGCAATCCTTA  F: CCTGTAGCCCACGTCGTAG  R: GGGAGTAGACAAGGTACAAC  F: TGCATCAACTTTGTGGCAAT  R: ATAGAGGCCGATTTCCTTGG  F: AAGGGCCATGGACTATTTCC  R: GACTCCACCCGATGACAGTT  F: CTCTCCAGGAGTCATCGTAG  R: CCAACACCAGCTGATACTTT  F: CCGGGATGGTGATCGGTTTT  R: CAGATGATCCGGGGCAATGA |  |
| *gapdh* | F: GGTTGTCTCCTGCGACTTCA | Inner reference used in qRT-PCRs |
|  | R: GGTCCAGGGTTTCTTACTCC |  |

F: forward primer. R: reverse primer.
